# The Reality of Medical Reality Television: Analysis of Physician Demographics, Trauma, and Cardiopulmonary Resuscitation (CPR) Characteristics

**DOI:** 10.7759/cureus.26610

**Published:** 2022-07-06

**Authors:** Brooke P Lichak, Robert P Olympia

**Affiliations:** 1 Internal Medicine, Penn State Health Milton S. Hershey Medical Center, Hershey, USA; 2 Emergency Medicine and Pediatrics, Penn State Health Milton S. Hershey Medical Center, Hershey, USA

**Keywords:** television, medical television, trauma, cpr, physician demographic, medical documentary, reality television

## Abstract

Background

Television and media have a profound effect on viewers’ understanding and interpretation of the world we live in. Reality television can be even more influential to viewers given its depiction of “real life”.

Materials and methods

Every episode (n=46) was analyzed from five medical reality television shows. *Hopkins, Boston Med, NY Med, Vanderbilt MDs, *and *Lenox Hill* were selected based on criteria requiring the show to be a reality show or docuseries that recorded unscripted patient interactions in the inpatient setting or emergency department.

Results

Of the 185 physicians shown on medical reality television, most were male (76.8%), white (80.0%), and surgeons (62.2%). Of the 417 patients shown on television, 72 patients had a traumatic mechanism of injury. Traumatic mechanisms included injury due to motor vehicle accident (29.2%), firearm (26.4%), cutting/piercing (12.5%), fall (12.5%), and fire/flame/hot substance (6.9%). Twenty-two of the 417 patients required cardiopulmonary resuscitation (CPR). Seven patients (31.8%) experienced cardiac arrest due to a traumatic mechanism of injury.

Conclusions

There was an overrepresentation of male physicians, white physicians, and surgeons on medical reality television compared to current demographic data on physicians (p<0.01). Traumatic mechanisms of injury by firearm, cutting/piercing, fire/flame/hot substance and traumatic causes of cardiac arrest were over-represented on television compared to current trauma and CPR registry data (p<0.01). This skewed “reality” of medicine as a non-diverse landscape riddled with trauma has the potential to profoundly impact viewers’ understanding of medical professionals and the medical field.

## Introduction

Cultivation theory, first described by George Gerbner in 1969, is the idea that exposure to media substantially influences viewers’ perceptions of their reality and blurs the lines between television reality and social reality [1]. Several studies have shown that network television has changed how viewers perceive the medical field and providers including perceived confidence in medicine, personality traits of medical providers, and mortality rates of illnesses [2-6]. Most importantly, studies have also shown that the more "real" a television show seems to viewers, the more it can change its viewers' perceptions about the world [7]. For this reason, television shows that are filmed without a script in real hospitals with real patients and providers have the potential to profoundly impact viewers’ perceptions of the medical field. 

While medical dramas have been analyzed to compare what is shown on television to real-world outcomes such as cardiopulmonary resuscitation (CPR) survival rates, there has never been a study analyzing the demographics of physicians, causes of illness, or CPR characteristics shown in medical reality television [8-11]. The objective of this study was to assess the accuracy of information being shown to the public about medicine through medical reality television.

## Materials and methods

Every episode (n=46) was analyzed from five medical reality television shows, *Hopkins*, *Boston Med*, *NY Med*, *Vanderbilt MDs*, and* Lenox Hill *(Table 1). The shows included in the analysis were characterized as reality shows or docuseries that recorded unscripted patient interactions in the inpatient setting or emergency department. Reality medical television shows that were re-enactments of patient stories or took place in the pre-hospital setting were excluded. Data was collected on all 185 physicians and 417 patients shown. Physician demographics (sex, race, and specialty), mechanism of injury from trauma, and CPR event characteristics (estimated age, sex, race of patient, underlying cause of arrest, and mortality rates) were recorded by one medical student. Data from the Association of American Medical Colleges, American College of Surgeons, Resuscitation Outcomes Consortium Registry, and National Registry of Cardiopulmonary Resuscitation were used for comparison given their large sample sizes in the tables below. A two-tailed t-test with a significance level of 0.05 was used to assess for statistical significance. The Institutional Review Board at Penn State Hershey Medical Center deemed the study exempt.

**Table 1:** Description of Medical Reality Television Shows *PG (parental guidance suggested), TV-14 (parents strongly cautioned), MA (mature audience only)

Television Show Name	Year Released	Rating*	Average Length of Episode (minutes)	Total # of Episodes Included in Analysis (n = 46)	Network	Description
Hopkins	2008	PG	43	7	ABC	Focuses on the doctors, patients, and families at Johns Hopkins Hospital
Boston Med	2010	PG	42	8	ABC	Documents the work of doctors, nurses, patients, and their families at three hospitals in Boston
NY Med	2012	PG	42	16	ABC	Follows the medical staff and patients at various hospitals in New York and New Jersey
Vanderbilt MDs	2014	TV-14	42	6	USA	Documents the lives and work of seven medical residents at Vanderbilt University Medical Center
Lenox Hill	2020	MA	50	9	Netflix	Follows the lives of four doctors and their patients in Lenox Hill Hospital in New York City

## Results

Data was collected on 185 physicians identified as an MD (Doctor of Medicine) or DO ( Doctor of Osteopathic Medicine) on television. While the total proportion of male and female physicians was portrayed accurately on television when compared to national demographic data on physicians, diversity with regards to physician race and specialty were grossly misrepresented. Most physicians shown were white and practiced a surgical specialty (Table 2).

**Table 2:** Demographics of Physicians on Medical Reality Television Compared to Reality *Includes - General Surgery, Interventional Cardiology, Neurological Surgery, Orthopedic Surgery, Plastic Surgery, Thoracic Surgery, Urology, Vascular and Interventional Radiology, and Vascular Surgery a - Association of American Medical Colleges. “Active Physicians by Sex and Specialty, 2017.” AAMC, Association of American Medical Colleges, December 2017, https://www.aamc.org/data-reports/workforce/interactive-data/active-physicians-sex-and-specialty-2017. Accessed 3 March 2022. b - Association of American Medical Colleges. “Percentage of all active physicians by race/ethnicity, 2018.” AAMC, Association of American Medical Colleges, 1 July 2019, https://www.aamc.org/data-reports/workforce/interactive-data/figure-18-percentage-all-active-physicians-race/ethnicity-2018. Accessed 3 March 2022.

	Medical Reality Television	Reality	P-value
Sex			
Male	142 (76.8%)	577,962^a^ (64.8%)	<0.01
Female	43 (23.2%)	313,808^a ^(35.2%)	<0.01
Total	185 (100%)	891,770^a^ (100%)	
Race			
White	148 (80.0%)	516,304^b^ (56.2%)	<0.01
African American	9 (4.9%)	45,534^b^ (5.0%)	0.95
Asian	28 (15.1%)	157,025^b^ (17.1%)	0.48
Other	0 (0.0%)	199,684^b^ (21.7%)	<0.01
Total	185 (100%)	918,547^b^ (100%)	
Specialty			
Non-Surgical Field	70 (37.8%)	809,815^a^ (90.8%)	<0.01
Surgical Field*	115 (62.2%)	81,955^a^ (9.2%)	<0.01
Total	185 (100%)	891,770^a^ (100%)	

Of the 417 patients shown on television, 72 suffered traumatic injuries. Traumas due to a mechanism of injury categorized as firearm, cut/pierce and fire/flame/hot object/substance were grossly over-represented on television while traumas due to a mechanism of injury categorized as falls were underrepresented (Table 3).

**Table 3:** Mechanism of Injury of Traumas Displayed on Medical Reality Television Versus Reality *Includes - Car versus person and car versus stationary object **Other - Physical abuse (n=5), Head Strike (n=1), Drowning (n=1), Plane Crash (n=1), Crush Injury (n=1) c - American College of Surgeons. “National Trauma Data Bank 2016 Annual Report.” National Trauma Data Bank, 2016, https://www.facs.org/-/media/files/quality-programs/trauma/ntdb/ntdb-annual-report-2016.ashx Accessed 3 March 2022.

Mechanism of Injury	Medical Reality Television	Reality	P-value
Motor Vehicle Accident*	21 (29.2%)	223,866^c^ (26.0%)	0.54
Firearm	19 (26.4%)	36,325^c ^(4.2%)	<0.01
Cut/Pierce	9 (12.5%)	35,565^c^ (4.1%)	<0.01
Fall	9 (12.5%)	380,800^c^ (44.2%)	<0.01
Fire/Flame/Hot Object/Substance	5 (6.9%)	16,278^c^ (1.9%)	<0.01
Other**	9 (12.5%)	169,054^c^ (19.6%)	0.13
Total	72 (100%)	861,888^c^ (100%)	

Of the 22 cardiac arrests shown on television, traumatic causes of cardiac arrest were over-represented while medical causes, including cardiac specific-pathology and electrolyte disturbances, were largely under-represented (Table 4).

**Table 4:** Demographics of Patients Requiring CPR and Outcomes of CPR in Medical Reality Television Versus Reality *Unknown - Patient identifiers blurred on television to protect patient privacy d - Blewer, Audrey L et al. “Gender Disparities Among Adult Recipients of Bystander Cardiopulmonary Resuscitation (CPR) in the Public.” Circulation. Cardiovascular quality and outcomes, vol. 11, no. 8, 2018, doi:10.1161/CIRCOUTCOMES.118.004710 e - Peberdy, Mary Ann et al. “Cardiopulmonary Resuscitation of Adults in the Hospital: A Report of 14,720 Cardiac Arrests from the National Registry of Cardiopulmonary Resuscitation.” Resuscitation, vol. 58, no. 3, 2003, pp. 297-308, doi:10.1016/s0300-9572(03)00215-6

	Medical Reality Television	Reality	P-value
Sex			
Male	13 (59.1%)	12,225^d^ (63.2%)	0.69
Female	9 (40.9%)	7,106^d^ (36.8%)	0.69
Total	22 (100%)	19,331^d ^(100%)	
Age			
Pediatric	2 (9.1%)	Data not available^d^	
Age 18-60	7 (31.8%)	7,554^d^ (39.1%)	0.48
Age 60+	3 (13.6%)	11,766^d^ (60.9%)	<0.01
Unknown*	10 (45.5%)	0^d^ (0.0%)	
Total	22 (100%)	19,320^d^ (100%)	
Race			
White	7 (31.8%)	6,349^d^ (32.8%)	0.92
African American	6 (27.3%)	3,148^d^ (16.3%)	0.16
Other or Unknown*	9 (40.9%)	9,834^d^ (50.9%)	0.35
Total	22 (100%)	19,331^d^ (100%)	
Cause of Arrest - Illness Category			
Trauma	7 (31.8%)	294^e^ (2.0%)	<0.01
Medical or Surgical	5 (22.7%)	14,279^e^ (97.0%)	<0.01
Unknown	10 (45.5%)	147^e^ (1.0%)	<0.01
Total	22 (100%)	14,720^e^ (100%)	
Mortality			
Survived	7 (31.8%)	6,477^e^ (44.0%)	0.25
Did Not Survive	14 (63.6%)	8,243^e^ (56.0%)	0.47
Unknown	1 (4.6%)	0^e^ (0.0%)	
Total	22 (100%)	14,720^e^ (100%)	

## Discussion

Medical television debuted over seven decades ago with *City Hospital* and has captivated millions of viewers through over one hundred different medical television series [12]. It was not until 1997 however, that medical reality television made its first appearance. Starting with *Trauma: Life in the E.R.*, there are now dozens of shows that capture medicine without perfect lighting, scripts, and flawlessly timed climaxes of patient stories that traditional medical dramas can afford. This seemingly raw and unfiltered peek behind the curtain gives viewers an intimate view of what the medical field truly looks like. Or is it?

While television depicts a seamless storyline, hundreds to thousands of hours of footage are taken for a single series and only a handful of hours make the final cut for broadcast. Since higher viewership equals larger profits, producers may have an incentive to show the “flashier” aspects of medicine including surgeries, trauma, and cardiac arrests with shocking outcomes. Physicians and patients may also choose to exhibit more dramatic emotions and facial expressions since they know they are being filmed. This may explain why so many surgeons, rare causes of traumas, and dramatic causes of cardiac arrest are disproportionally shown on television. While the decision to include these patient cases over more mundane cases such as high blood pressure management and treatment of the common cold is rather unsurprising, the decision to film a largely homogenous cast of white male physicians is intriguing and warrants further reflection. 

Data from the Association of American Medical Colleges shows that there are far more female and non-white physicians than are accounted for on medical reality television (Table 2), so why are there so few represented? Each show was filmed in a large metropolitan area (i.e. Boston, New York City, Baltimore, Nashville) with high demographic diversity. One might expect that the physician workforce at these hospitals would reflect this diversity, but that is not the case. One explanation may be that the hospitals represented are some of the best in the nation and therefore attract physicians with high accolades. These are often physicians who come from prestigious schools and those with access to more research and academic opportunities beginning as early as grade school. Given these schools and opportunities can require significant sums of money, those from more affluent backgrounds can more easily advance their careers and gain employment at these world-renowned hospital systems. While reality television may initially appear as an unaltered reflection of society, there are many decisions that the film crew, producers, physicians, and patients make that distort this view. When our perception of reality does not fit the reality presented in the media, a new, slightly modified reality is constructed to improve this cognitive dissonance. This may not only lead to skewed perceptions of medicine but also what a typical physician looks like. 

There are several limitations to this study. A single viewer was responsible for collecting and categorizing the data. Additionally, the data collected regarding patient and provider demographics, including age, race, and ethnicity, is largely subjective which could lead to possible misclassification and subsequent measurement bias. Due to the limited number of medical reality television shows that have aired, there was a small sample size of traumas and cardiac arrests, making it challenging to draw statistically significant conclusions. Lastly, all shows were filmed in the United States, so results may not be generalizable to other countries, especially those with different healthcare system models, training requirements, and population demographics. Future research that measures the impact of medical reality television on viewers’ perception of diversity in the medical field and expected patient outcomes would provide particularly valuable information regarding just how influential these shows can be. 

## Conclusions

Our study shows that medical reality television can portray a distorted picture of medicine in which many patients who present to the hospital have a traumatic injury, are managed by white male surgeons, and are often on the brink of death. While this dramatization of medicine certainly leads to more viewers and higher ratings, it does not accurately portray the medical field. This marked divergence from reality regarding physician workforce diversity, trauma, and mechanisms of cardiac arrest not only leads to a skewed reality of medicine for viewers but can also profoundly influence young viewers’ career choices, as their diverse backgrounds and interests are not represented by the narrow picture of medicine and medical professionals on television.
